# Characteristics and outcomes of pregnant women admitted to hospital with confirmed SARS-CoV-2 infection in UK: national population based cohort study

**DOI:** 10.1136/bmj.m2107

**Published:** 2020-06-08

**Authors:** Marian Knight, Kathryn Bunch, Nicola Vousden, Edward Morris, Nigel Simpson, Chris Gale, Patrick O’Brien, Maria Quigley, Peter Brocklehurst, Jennifer J Kurinczuk

**Affiliations:** 1 National Perinatal Epidemiology Unit, Nuffield Department of Population Health, University of Oxford, Oxford OX3 7LF, UK; 2 Faculty of Life Sciences and Medicine, King’s College London, London, UK; 3 Norfolk and Norwich University Hospital, Norwich, UK; 4 Department of Women's and Children's Health, School of Medicine, University of Leeds, Leeds, UK; 5 Neonatal Medicine, School of Public Health, Faculty of Medicine, Imperial College London, London, UK; 6 Institute for Women’s Health, University College London, London, UK; 7 Birmingham Clinical Trials Unit, Institute of Applied Health Research, University of Birmingham, Birmingham, UK

## Abstract

**Objectives:**

To describe a national cohort of pregnant women admitted to hospital with severe acute respiratory syndrome coronavirus 2 (SARS-CoV-2) infection in the UK, identify factors associated with infection, and describe outcomes, including transmission of infection, for mothers and infants.

**Design:**

Prospective national population based cohort study using the UK Obstetric Surveillance System (UKOSS).

**Setting:**

All 194 obstetric units in the UK.

**Participants:**

427 pregnant women admitted to hospital with confirmed SARS-CoV-2 infection between 1 March 2020 and 14 April 2020.

**Main outcome measures:**

Incidence of maternal hospital admission and infant infection. Rates of maternal death, level 3 critical care unit admission, fetal loss, caesarean birth, preterm birth, stillbirth, early neonatal death, and neonatal unit admission.

**Results:**

The estimated incidence of admission to hospital with confirmed SARS-CoV-2 infection in pregnancy was 4.9 (95% confidence interval 4.5 to 5.4) per 1000 maternities. 233 (56%) pregnant women admitted to hospital with SARS-CoV-2 infection in pregnancy were from black or other ethnic minority groups, 281 (69%) were overweight or obese, 175 (41%) were aged 35 or over, and 145 (34%) had pre-existing comorbidities. 266 (62%) women gave birth or had a pregnancy loss; 196 (73%) gave birth at term. Forty one (10%) women admitted to hospital needed respiratory support, and five (1%) women died. Twelve (5%) of 265 infants tested positive for SARS-CoV-2 RNA, six of them within the first 12 hours after birth.

**Conclusions:**

Most pregnant women admitted to hospital with SARS-CoV-2 infection were in the late second or third trimester, supporting guidance for continued social distancing measures in later pregnancy. Most had good outcomes, and transmission of SARS-CoV-2 to infants was uncommon. The high proportion of women from black or minority ethnic groups admitted with infection needs urgent investigation and explanation.

**Study registration:**

ISRCTN 40092247.

## Introduction

The World Health Organization declared a global pandemic of coronavirus disease 2019 (covid-19) caused by severe acute respiratory syndrome coronavirus 2 (SARS-CoV-2) in March 2020.^1^ As the number of confirmed cases increases, evidence on the transmission, incidence, and effect of SARS-CoV-2 infection in mothers and their babies remains limited. Pregnant women are not thought to be more susceptible to the infection than the general population.^2^ ^3^ However, changes to the immune system mean that pregnant women may be more vulnerable to severe infection.^4^ Evidence from other similar viral illnesses, such as influenza A/H1N1,^5^ ^6^ ^7^ ^8^ severe acute respiratory syndrome,^9^ and Middle East respiratory syndrome,^10^ ^11^ suggest that pregnant women are at greater risk of severe maternal and neonatal morbidity and mortality. Some evidence suggests that the risk of critical illness may be greatest in the later stages of pregnancy.^5^ ^10^ ^11^

To the best of our knowledge, as of 12 May 2020 more than 90 scientific reports of SARS-CoV-2 infection in pregnancy had been published in English,^2^ ^10^ ^12^ ^13^ none of which was population based. Most reported cases occurred in the third trimester, and around half of women gave birth during the acute infection episode. Most women were delivered by caesarean section, predominantly for maternal indication, although at least three studies reported cases of fetal distress.^13^ ^14^ ^15^ ^16^ ^17^ Most women developed mild or moderate symptoms including cough, fever, and breathlessness, and only a small number developed severe disease.^15^ ^16^ ^18^ ^19^ ^20^ ^21^ Risk factors are suggested to mirror those in the general population, with a high proportion of women with severe covid-19 having a raised body mass index or comorbidities such as pulmonary conditions (25%) or pre-existing cardiac disease (17%).^15^ ^17^

Evidence suggests that severe covid-19 in pregnancy is associated with iatrogenic preterm delivery (75%), predominantly for maternal indication and in the third trimester.^17^ Most neonates born to mothers with confirmed SARS-CoV-2 infection were asymptomatic and discharged home well. A small number of neonates had symptoms, with a minority needing admission to neonatal specialist care^14^ ^15^; only in a few instances have neonates had positive tests for SARS-CoV-2 following delivery.^22^ ^23^ ^24^ ^25^ Three neonates had elevated serum IgM antibodies identified shortly after birth in umbilical blood, but SARS-CoV-2 was not identified in any of these infants in the neonatal period despite testing.^23^ ^25^ These three infants had no symptoms, so the significance of vertical transmission remains unknown.

The aim of this study was to describe, on a population basis, characteristics and outcomes of pregnant women admitted to hospital with SARS-CoV-2 in the UK, in order to inform ongoing guidance and management. This study was designed in 2012 and hibernated pending a pandemic; it was activated by the UK Department of Health and Social Care as an urgent public health study in response to the SARS-CoV-2 pandemic.

## Methods

We did a national prospective observational cohort study using the UK Obstetric Surveillance System (UKOSS).^26^ UKOSS is a research platform that collects national population based information about specific severe complications of pregnancy from all 194 hospitals in the UK with a consultant led maternity unit. We asked nominated reporting clinicians to notify us of all pregnant women with confirmed SARS-CoV-2 infection admitted to their hospital, using a live reporting link specific to each individual reporter. For the purposes of this study, we defined confirmed maternal infection as detection of viral RNA on polymerase chain reaction testing of blood or a nasopharyngeal swab, respiratory compromise in the presence of characteristic radiographic changes of covid-19, or both. At the time covered by the study, women were tested only if they had symptoms of SARS-CoV-2 infection. We defined neonatal infection as detection of viral RNA on polymerase chain reaction testing of blood or a nasopharyngeal swab or aspirate. The process of data collection was enabled by research midwives and nurses from the UK’s National Institute of Health Research Clinical Research Network following its adoption as an urgent public health priority study.^27^ In addition, we sent nominated clinicians a reporting email at the end of the month to ensure that all cases had been reported and to confirm zero reports (active negative surveillance). After notification, we asked clinicians to complete an electronic data collection form containing details of each woman’s characteristics, management, and outcomes. Reporters who had not returned data were contacted by email at weeks one, two, and three after notification. This analysis reports characteristics and outcomes of women who were notified as admitted to hospital between 1 March and 14 April 2020 and for whom complete data had been received by 29 April 2020.

We defined body mass index on the basis of the first recorded weight in pregnancy and gestational age according to the final estimated date of delivery based on ultrasound assessment. Ethnic group was based on women’s self-report, as recorded in medical records. We cross checked data on maternal and perinatal deaths with data from the MBRRACE-UK collaboration, the organisation responsible for maternal and perinatal death surveillance in the UK.^28^

### Sample size and statistical analysis

In this national observational study, the study sample size was governed by the disease incidence, so we did no formal power calculation. We calculated the incidence of admission to hospital with confirmed SARS-CoV-2 infection in pregnancy and among population subgroups by using denominator estimates based on the most recently available (2018) national maternity data for the constituent countries of the UK and National Maternity and Perinatal Audit data from 2016-17 for body mass index groups. We present numbers, proportions, and risk ratios with 95% confidence intervals. Continuous data are summarised as medians with interquartile ranges. We did a sensitivity analysis excluding women from London, the West Midlands, and the North West of England to explore the proportion of women from black and minority ethnic groups admitted with SARS-CoV-2 in pregnancy outside of the major urban centres. We used Stata version 15 for statistical tabulation and analyses.

### Study registration

The study is registered with ISRCTN, number 40092247, and is still open to case notification. The study protocol is available at https://www.npeu.ox.ac.uk/ukoss/current-surveillance/covid-19-in-pregnancy.

### Patient and public involvement

Patients and the public were involved in the design of the study, and, as part of the UKOSS Steering Committee, in the conduct of the study and interpretation of the result.

## Results

We received responses from all 194 hospitals with obstetric units in the UK. From 1 March to 14 April 2020, 630 women admitted to hospital with confirmed SARS-CoV-2 infection in pregnancy were notified in the UK, among an estimated 86 293 maternities. Data were returned for 579 (92%) women; 15 were duplicate cases, 35 were reported in error, 87 had the diagnosis made as outpatients and were not admitted overnight, nine had no positive polymerase chain reaction test and no evidence of pneumonitis on imaging, and six had no evidence of infection during pregnancy, leaving 427 pregnant women admitted to hospital with confirmed SARS-CoV-2 across the UK. This represents an estimated incidence of hospital admission of 4.9 (95% confidence interval 4.5 to 5.4) pregnant women per 1000 maternities.

**Fig 1:**
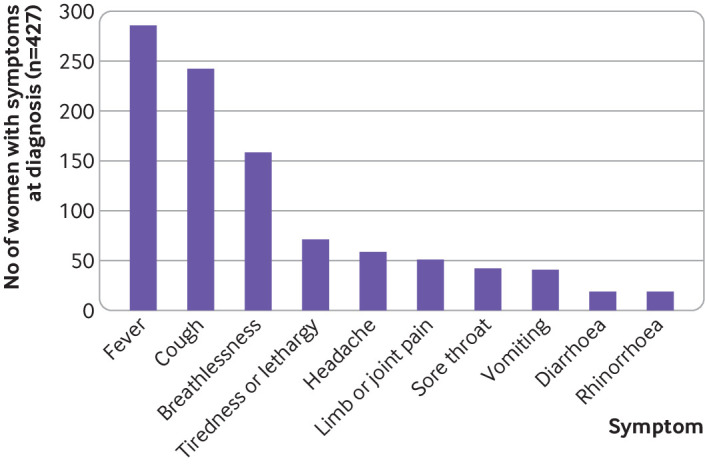
Maternal symptoms at diagnosis of covid-19

Women had symptoms at a median of 34 (interquartile range 29-38) completed weeks’ gestation, with most women admitted to hospital having symptoms in the third trimester of pregnancy or peripartum (342/424; 81%). The most common symptoms reported by women were fever, cough, and breathlessness (fig 1). Table 1 shows the characteristics of the women. In the sensitivity analysis excluding women from London, the West Midlands, and the north west of England, 75 (46%) of 162 women admitted were from black and minority ethnic groups. The incidence of admission with confirmed SARS-CoV-2 infection in pregnancy seemed to vary according to women’s ethnic group, age, and body mass index (table 2).

**Table 1:** Characteristics of pregnant women with confirmed SARS-CoV-2 infection for whom data were available, UK, 1 March to 14 April 2020

Characteristic	No (%)* of women (n=427)
Age, years:	
<20	4 (1)
20-34	248 (58)
≥35	175 (41)
Body mass index:	
Normal	126 (31)
Overweight	141 (35)
Obese	140 (34)
Missing data	20
Woman and/or partner in paid work	343 (80)
Black or other minority ethnic group (all)	233 (56)
Asian	103 (25)
Black	90 (22)
Chinese/other	30 (7)
Mixed	10 (2)
Missing data	10
Current smoking	20 (5)
Missing data	8
Pre-existing medical problems	145 (34)
Asthma	31 (7)
Hypertension	12 (3)
Cardiac disease	6 (1)
Diabetes	13 (3)
Multiparous	263 (62)
Missing data	4
Multiple pregnancy	8 (2)
Gestational diabetes	50 (12)
Gestation at symptom onset, weeks:	
<22	22 (5)
22-27	60 (14)
28-31	64 (15)
32-36	106 (25)
≥37	142 (33)
Peripartum	30 (7)
Missing data	3

* Percentages of those with complete data.

**Table 2:** Estimated incidence of admission with SARS-CoV-2 infection in pregnancy among different population subgroups

Characteristic	Estimated No of maternities	No of pregnant women admitted with SARS-CoV-2	Incidence per 1000 maternities	Rate ratio (95% CI)
Age*, years:				
<20	2532	4	1.6	0.4 (0.1 to 1.1)
20-34	63 768	248	3.9	1 (reference)
≥35	19 992	175	8.8	2.3 (1.8 to 2.7)
Body mass index†:				
Normal (<25)	36 377	126	3.5	1 (reference)
Overweight (25 to <30)	20 836	141	6.8	2.0 (1.5 to 2.5)
Obese (≥30)	16 154	140	8.7	2.5 (2.0 to 3.2)
Ethnic group (England only)‡:				
White	49 282	173	3.5	1 (reference)
Asian	7400	103	13.9	4.0 (3.1 to 5.1)
Black	3135	89	28.4	8.1 (6.2 to 10.5)
Chinese/other	2960	28	9.5	2.7 (1.7 to 4.0)
Mixed	1304	9	6.9	2.0 (0.9 to 3.8)

* Estimated number of maternities based on number of maternities in UK occurring during March and 14/30 of April 2018. Four women with unknown age excluded from denominator.

† Estimated number of maternities based on number of maternities in GB during March and 14/30 of April in year April 2016 to 31 March 2017. Women with unknown body mass index excluded from both numerator (20) and denominator (12 291).

‡ Estimated number of maternities based on number of maternities in England occurring during March and 14/30 of April 2018. Women with unknown ethnicity excluded from both numerator (10) and denominator (7996).

Two hundred and sixty six (62%) women admitted to hospital gave birth or had a pregnancy loss; the remaining 161 (38%) women had ongoing pregnancies at the time of this analysis. Forty one (10%) women needed level 3 critical care; four of these women received extracorporeal membrane oxygenation (table 3). Of the women who received critical care, 33 (80%) had been delivered, 27 (66%) of them owing to worsening respiratory condition; eight (20%) were still pregnant. All eight (100%) of the women who were still pregnant after their critical care admission had been discharged. Nineteen (58%) of the 33 postnatal women had been discharged at the time of this analysis; three women admitted to critical care had died, and 11 (33%) were still inpatients, of whom seven (64%) remained in critical care. Overall, five women who were admitted with confirmed SARS-CoV-2 died, a case fatality of 1.2% (95% confidence interval 0.4% to 2.7%) and a SARS-CoV-2 associated maternal mortality rate of 5.8 (1.9 to 13.5) per 100 000 maternities. Three women died as a direct result of complications of covid-19 and two from other causes. In total, 25 (6%) women, 7 (28%) antenatal and 18 (72%) postnatal, were still inpatients at the time of this analysis.

**Table 3:** Hospital outcomes and diagnoses among women with confirmed SARS-CoV-2 infection in pregnancy

Maternal outcomes	No (%) of women (n=427)
Needed critical care	41 (10)
Needed extracorporeal membrane oxygenation	4 (1)
SARS-CoV-2 pneumonia on imaging	104 (24)
Final outcome:	
Died	5 (1)
Discharged well	397 (93)
Still in hospital	25 (6)

Nine (2%) women were treated with an antiviral agent. Eight of them were given oseltamivir, one of whom also received lopinavir/ritonavir. One woman was given remdesivir. All women managed with antivirals were discharged home. Sixty four (15%) women were given corticosteroids for fetal lung maturation, of whom 47 (73%) had given birth. Thirteen (20%) of these 64 women remained as inpatients, 12 (92%) of whom had given birth.

Four women (0.9% of those admitted; 4.6 (1.3 to 11.2) per 100 000 maternities) had a miscarriage, at a range of 10 to 19 weeks’ gestation. Of the 262 women who had given birth, 196 (75%) gave birth at term (table 4). Sixty six women gave birth preterm; 53 (80%) had iatrogenic preterm births, 32 (48%) due to maternal covid-19, nine (14%) due to fetal compromise, and 12 (18%) due to other obstetric conditions. Fifty nine per cent of women (n=156) had a caesarean delivery, but most of the caesarean births occurred for indications other than maternal compromise due to SARS-CoV-2 infection. Forty two women (27% of those who had a caesarean birth) had a caesarean birth for reasons of maternal compromise, 37 (24%) due to concerns about fetal compromise, 30 (19%) due to failure to progress in labour or failed induction of labour, 25 (16%) for other obstetric reasons, 16 (10%) because of previous caesarean birth, and 6 (4%) at maternal request. Twenty nine (19%) women had general anaesthesia for their caesarean birth; 18 (62%) of these women were intubated because of maternal respiratory compromise, and 11 (38%) were intubated to allow for urgent delivery.

**Table 4:** Pregnancy and infant outcomes among pregnant women with confirmed SARS-CoV-2 infection

Pregnancy outcomes	No (%) of women (n=427)
Ongoing pregnancy	161 (38)
Pregnancy completed	266 (62)
Pregnancy loss	4 (1)
Stillbirth	3 (1)
Live birth (including six women who gave birth to twins)	259 (97)
Neonatal death	2 (1)
Gestation at end of pregnancy, weeks:	
<22	4 (2)
22-27	6 (2)
28-31	17 (6)
32-36	43 (16)
≥37	196 (74)
Median (interquartile range)	38 (36-40)
Mode of birth*:	
Caesarean, maternal indication due to SARS-CoV-2	42 (16)
Caesarean, other indication	114 (44)
Operative vaginal	28 (11)
Unassisted vaginal	78 (30)

* Excluding four women with pregnancy losses.

Five babies died; three were stillborn and two died in the neonatal period. Three deaths were unrelated to SARS-CoV-2 infection and were due to obstetric conditions unrelated to SARS-CoV-2 infection and/or pre-existing fetal conditions; for two stillbirths, whether SARS-CoV-2 contributed to the death was unclear. Sixty seven (25%) of 265 liveborn infants were admitted to a neonatal unit, 50 (75%) of whom were preterm, including 23 (34%) who were less than 32 weeks’ gestation (table 5). One infant was diagnosed as having neonatal encephalopathy (grade 1) after a spontaneous vaginal birth at term. Twelve (5%) infants of women admitted to hospital with infection tested positive for SARS-CoV-2 RNA, six of them within the first 12 hours after birth. Two of the six infants with early onset SARS-CoV-2 infection were from unassisted vaginal births; four were born by caesarean, three of which were pre-labour. No viral analyses were performed on umbilical cord blood, placenta, or vaginal secretions. The six infants who developed later infection were born by pre-labour caesarean (n=4) and vaginal birth (n=2). Only one of the infants with an early positive test for SARS-CoV-2 RNA was admitted to a neonatal unit, compared with five infants with a later positive test.

**Table 5:** Infant outcomes among liveborn babies of women with confirmed SARS-CoV-2 infection in pregnancy

Infant outcomes	No (%) of liveborn infants of women with SARS-CoV-2 (n=265)*
Neonatal unit admission	67 (25)
Positive SARS-CoV-2 test (liveborn infants only):	
No	253 (95)
Positive test <12 hours of age	6 (2)
Positive test ≥12 hours of age	6 (2)

* Includes six sets of twins.

## Discussion

The clinical data from this national surveillance study show that one in 10 pregnant women admitted to hospital in the UK with SARS-CoV-2 infection needed respiratory support in a critical care setting, and one in 100 died. More than half of pregnant women admitted to hospital with SARS-CoV-2 infection in pregnancy were from black or other ethnic minority groups, 70% were overweight or obese, 40% were aged 35 or over, and a third had pre-existing comorbidities. More than half of all women admitted with SARS-CoV-2 infection had given birth at the time of the analysis; 12% were delivered preterm solely because of maternal respiratory compromise. Almost 60% of women gave birth by caesarean section; most caesarean births were for indications other than maternal compromise due to SARS-CoV-2 infection. One in 20 of the babies of mothers admitted to hospital subsequently had a positive test for SARS-CoV-2; half had infection diagnosed on samples taken at less than 12 hours after birth.

### Strengths and limitations of study

A major strength of this study is the design using the population based UKOSS research platform and thus the identification of a comprehensive, national cohort of infected pregnant women with high case ascertainment across all obstetric units in the UK. However, this rapid report has been produced at a time when active transmission of SARS-CoV-2 is still occurring, with around 100 pregnant women admitted to hospital in the UK with infection each week, and the limitations of these data must therefore be recognised. We do not yet have complete pregnancy outcomes for women who were admitted but subsequently discharged well, and several women were still inpatients at the time of writing. The data collected for this rapid national cohort study were restricted to essential items, so we do not have daily indicators of women’s clinical condition or results of blood and other tests. We sought to collect national, population based information on severe SARS-CoV-2 infection, defined as hospital admission, to capture the incidence and outcomes of severe disease in pregnancy. This study does not therefore provide any information about overall infection rates or the possibility of asymptomatic infection. Nevertheless, this study shows the strength of systems such as UKOSS, which can be rapidly activated to do comprehensive population based studies such as this in a public health emergency. UKOSS studies were activated for influenza A/H1N1 and Zika virus in pregnancy^29^ ^30^; countries in the International Network of Obstetric Survey Systems (INOSS)^31^ are also doing similar national studies to allow for the unification of population based data across multiple countries and avoiding the biases of data collected through centre based registries. The National Institute for Health Research’s Clinical Research Network,^32^ with midwifery and obstetric leads coordinating networks of research staff, was another strength of this study, helping to ensure rapid and accurate collection of these valuable data even in the context of the pressurised health system in a pandemic. UKOSS is the only national research platform in the UK for conducting such studies, and it should be noted that all other reports of women admitted to hospital with SARS-CoV-2 in pregnancy in the UK will be subsets of UKOSS data.

### Comparison with other studies

The addition of these national, population based data to existing reports provides clarity on the outcomes of infection in pregnant women. Previous published information has been largely based on case series from individual hospitals or cases identified across small series of hospitals but with a lack of clarity about the proportion of cases ascertained, with problems of overlap and duplicate reporting; population based data are essential to provide unbiased information on incidence and outcomes. During the period when these data were collected, around 90 000 women gave birth in the UK; 427 were notified as having been admitted with SARS-CoV-2 in pregnancy—fewer than one woman admitted for every 200 women giving birth. Approximately one woman per 2400 giving birth needed critical care admission. The overall maternal mortality rate with confirmed SARS-CoV-2 infection was around one in 18 000 women giving birth. The rates of critical care unit admission and mortality among pregnant women admitted to hospital with SARS-CoV-2 infection are comparable to the rates among the general population of women of reproductive age admitted to UK hospitals with infection, of whom 20-35% receive critical care and 1-4% die.^33^

The high proportion of women from black and other minority ethnic groups admitted to hospital with SARS-CoV-2 in pregnancy is of concern and should be investigated further. Our sensitivity analysis suggests that this cannot simply be explained by a higher incidence in the main metropolitan areas with higher proportions of women from ethnic minority groups, as the high proportion remained when we excluded women from London, the West Midlands, and the north west of England. Ethnic disparities in incidence and outcomes have been noted among non-pregnant populations with SARS-CoV-2 infection, notably in the US,^34^ and various possible reasons have been suggested for these observed disparities, including social behaviours, health behaviours, comorbidities, and potentially genetic influences.^35^ It should be noted that over-representation of ethnic minority and other groups among the cohort of pregnant women admitted with SARS-CoV-2 infection may reflect a higher risk of infection, a higher risk of severe disease given infection among vulnerable subgroups, or both. Health system factors have been suggested to underlie the disparity in the US; the fact that these disparities exist in a country with a universal free to access healthcare system indicate that the health system cannot be the sole explanation.

In common with previous reports, most women admitted to hospital with SARS-CoV-2 infection in pregnancy were in the late second or third trimester, which replicates the pattern seen for other respiratory viruses with women in later pregnancy being more severely affected. This supports the current guidance for strict social distancing measures among pregnant women, particularly in their third trimester.^2^ It should be noted, however, that higher hospital admission rates in the third trimester were also reported in the context of influenza,^36^ and thought to be for precautionary reasons, rather than necessarily because of maternal compromise. Although case notification has been augmented through a link with the UK Early Pregnancy Surveillance System (UKEPSS),^37^ the route of identification of the women included in this series, through UK obstetric units, could also have led to under-ascertainment of women admitted in the early stages of pregnancy.

Outcomes for infants are largely reassuring when considering potential effects of SARS-CoV-2 infection acquired before or during birth; the small number of early polymerase chain reaction positive infants of mothers with infection did not have evidence of severe illness. This observation of only mild disease has also been reflected in early case reports of infant infection in the perinatal period.^22^ ^23^ ^24^ ^25^ Nevertheless, 2% of infants did have evidence of viral RNA in a sample taken within 12 hours of birth, which suggests that mother-to-infant viral transmission may be occurring. We have no evidence as to whether IgM was raised in these infants or whether viral transmission occurred in utero, during delivery via an infected birth canal, or postpartum via respiratory droplets, skin-to-skin contact, or breast feeding, but three infants had a positive test for SARS-CoV-2 following pre-labour caesarean section. We do not have information on whether these infants were isolated from the mother immediately after delivery, nor whether skin-to-skin contact was permitted. Using a recently suggested classification system,^38^ we therefore do not have sufficient evidence to suggest that these were congenitally acquired infections; they should be classified as possible neonatally acquired infections. During the study period, UK guidance for postnatal management of infants born to mothers with confirmed or suspected SARS-CoV-2 infection was to keep mother and infant together and to encourage breast feeding with consideration of using a fluid resistant surgical face mask for the mother. These findings emphasise the importance of infection control measures around the time of birth and support the advice given by WHO around precautions to take while breast feeding.

We did this study in a high resource setting with universal healthcare free at the point of access, and findings would therefore be generalisable to similar settings. The fact that most women experience mild infection would suggest that outcomes are likely to be good in settings with less well developed health systems. However, given the proportion of women admitted who needed critical care, the outcomes of severe infection will probably be poorer in the absence of such facilities.

### Conclusions

In the context of the covid-19 pandemic, ongoing collection of data on the outcomes of infection during pregnancy will remain important. Unanswered questions remain about the extent and effect of asymptomatic or mild infection. Serological studies, as well as those using retrospective data to identify women with either confirmed or presumed mild infection in pregnancy, will be essential to fully assess potential effects such as congenital anomalies, miscarriage, or intrauterine fetal growth restriction. Nevertheless, these data suggest that most women do not have severe illness and that transmission of infection to infants of infected mothers can occur but is uncommon.

**What is already known on this topic**

Published evidence on transmission, incidence, and effect of SARS-CoV-2 infection in mothers and their babies remains limited mainly to reports of single cases or small case series
Evidence from other similar viral illnesses suggest that pregnant women are at greater risk of severe maternal and neonatal morbidity and mortality
Cases of transmission of SARS-CoV-2 infection to the neonate have been reported, but how frequent this is on a population basis is unclear

**What this study adds**

More than half of pregnant women admitted to hospital with SARS-CoV-2 infection in pregnancy were from black or other ethnic minority groups
Most women did not have severe illness, and most were admitted in the third trimester of pregnancy
Transmission of infection to infants of infected mothers may occur but is uncommon

## Notes

Ethical approval: This study was approved by the HRA NRES Committee East Midlands – Nottingham 1 (reference 12/EM/0365).

Data sharing: Data from this study will be shared according to the National Perinatal Epidemiology Unit Data Sharing Policy available at https://www.npeu.ox.ac.uk/downloads/files/npeu/policies/Data%20Sharing%20Policy.pdf

The lead author (the manuscript’s guarantor) affirms that the manuscript is an honest, accurate, and transparent account of the study being reported; that no important aspects of the study have been omitted; and that any discrepancies from the study as planned (and, if relevant, registered) have been explained.

Dissemination to participants and related patient and public communities: Dissemination to participants is not possible as this study collected anonymous data only. Dissemination to women, families, and healthcare practitioners will be undertaken via social media and the programme website (https://www.npeu.ox.ac.uk/ukoss/publications-ukoss) and through summary articles for professional and third sector organisations.
